# Characterization of pediatric eosinophilic gastrointestinal disorders beyond eosinophilic esophagitis in a nationwide cohort

**DOI:** 10.1002/jpn3.70282

**Published:** 2025-11-19

**Authors:** Sara Renzo, Luca Scarallo, Selene Del Vespa, Giulia Angelino, Matteo Bramuzzo, Enrico Felici, Flavio Labriola, Francesca Laganà, Lorenzo Norsa, Salvatore Oliva, Marisa Piccirillo, Elena Pozzi, Francesca Rea, Giusy Russo, Francesco Valitutti, Giovanna Zuin, Paolo Lionetti

**Affiliations:** ^1^ Gastroenterology and Nutrition Unit, Meyer Children's Hospital IRCCS Florence Italy; ^2^ Department NEUROFARBA University of Florence Florence Italy; ^3^ Gastroenterology and Nutrition Unit, Bambino Gesu’ Children's Hospital, IRCCS Rome Italy; ^4^ Gastroenterology, Digestive Endoscopy and Nutrition Unit, Institute for Maternal and Child Health‐IRCCS “Burlo Garofolo,” Trieste Italy; ^5^ Pediatric and Pediatric Emergency Unit, “Umberto Bosio” Center for Digestive Diseases, The Children Hospital, AO SS Antonio e Biagio e Cesare Arrigo Alessandria Italy; ^6^ Pediatric Gastroenterology Unit, Maggiore Hospital Bologna Italy; ^7^ Pediatric Gastroenterology and Cystic Fibrosis Unit, Department of Human Pathology in Adulthood and Childhood “G. Barresi” University of Messina Messina Italy; ^8^ Pediatric Department, Children's Hospital Vittore Buzzi University of Milan Milan Italy; ^9^ Pediatric Hepatology Gastroenterology and Transplantation, ASST Papa Giovanni XXIII Bergamo Italy; ^10^ Maternal and Child Health Department, Pediatric Gastroenterology and Liver Unit Sapienza ‐ University of Rome Rome Italy; ^11^ Sapienza University of Rome, NESMOS Department, Pediatric Unit Sant'Andrea University Hospital Rome Italy; ^12^ Pediatric Section, Department of Medicine and Surgery University of Perugia Perugia Italy; ^13^ Pediatric Department Fondazione IRCCS San Gerardo dei Tintori Monza Italy

**Keywords:** budesonide, eosinophilia, very early onset‐EGIDs

## Abstract

**Objectives:**

Nonesophageal eosinophilic gastrointestinal disorders (non‐EoE EGIDs) are rare, underrecognized inflammatory diseases of the gastrointestinal (GI) tract, especially in children. Their clinical heterogeneity and lack of specific biomarkers contribute to diagnostic delays and therapeutic challenges.

**Methods:**

This retrospective multicenter study included pediatric patients (<18 years) diagnosed with non‐EoE EGIDs across 12 Italian centers affiliated with the Italian Society of Pediatric Gastroenterology Hepatology and Nutrition (SIGENP). The data were retrospectively collected from January 2012 to December 2022. Diagnosis was based on ESPGHAN histological criteria. Clinical, laboratory, endoscopic, and histological data were collected at baseline and during follow‐up. Treatment modalities and outcomes were analyzed.

**Results:**

A total of 86 patients (71% male; median age 10.5 years) were included. The stomach was the most commonly involved site (45.4%), followed by the small bowel (40.7%) and colon (24.4%). Multiple segment involvement occurred in 45.4% of cases. Abdominal pain (64.9%) and diarrhea (38.2%) were the most common symptoms, with diarrhea significantly associated with colonic involvement. Laboratory findings showed peripheral eosinophilia in 50% of patients and hypoalbuminemia in those with multisegment involvement. Very early‐onset EGIDs (>1 and <2 years) were associated with features such as predominant colonic involvement and anemia. Treatment responses varied: 50% achieved clinical remission after first‐line therapy (proton pump inhibitors, topical/systemic steroids, or elimination diets). Among 61/86 patients who underwent endoscopic follow‐up, 49.2% achieved macroscopic remission and 40.9% histological remission.

**Conclusions:**

Pediatric non‐EoE EGIDs present with heterogeneous symptoms and variable tract involvement. Younger children show distinct phenotypes. Laboratory findings are nonspecific and have limited diagnostic utility. Treatment remains challenging, with suboptimal response rates. These findings underscore the need for prospective studies.

## Abstract (highlights)

**What is Known:**

- Non‐EoE eosinophilic gastrointestinal disorders (non‐EoE EGIDs) are rare and underrecognized, particularly in pediatric populations.
- Clinical presentation is heterogeneous, nonspecific, often leading to diagnostic delays.
- Laboratory findings are nondiagnostic and endoscopic features lack specificity.
- Recent guidelines ESPGHAN/NASPGHAN suggest diagnostic and treatment strategies for pediatric population.

**What is New:**

- This pediatric cohort of non‐EoE EGIDs describes real‐world diagnostic and therapeutic approaches.
- EoE co‐occurrs in a significant proportion of patients, including those with isolated eosinophilic colitis.
- Very early‐onset cases (<2 years) show distinct patterns: predominant colonic involvement, anemia, and hypoalbuminemia.
- A substantial proportion of patients did not achieve clinical and/or histological remission after first‐line therapy, highlighting unmet treatment needs.

## 1 INTRODUCTION

Eosinophilic gastrointestinal disorders (EGIDs) beyond eosinophilic esophagitis (non‐EoE EGIDs) are rare conditions characterized by inflammation localized to segments of the gastrointestinal (GI) tract beyond the esophagus.^1^ These disorders are classified based on the anatomical location of the pathological eosinophilic infiltration. A standardized classification system includes terms such as eosinophilic gastritis (EoG), eosinophilic duodenitis (EoD), eosinophilic enteritis (EoN), which can be further subdivided into EoD, eosinophilic jejunitis (EoJ), and eosinophilic ileitis (EoI), as well as eosinophilic colitis (EoC), depending on the affected GI segment. If at least two GI segments are involved, the nomenclature reflects all affected areas.^2^, ^3^, ^4^

Clinical experience with non‐EoE EGIDs is limited, and comprehensive literature on these conditions, particularly in the pediatric population, is scarce. Diagnosis remains challenging due to the heterogeneous clinical presentation and the absence of definitive biological markers, often resulting in delays in diagnosis.^2^, ^5^ Clinical manifestations are nonspecific and vary based on the localization and depth of the inflammatory infiltrate. The most common presentation is predominantly mucosal disease, with patients typically experiencing abdominal pain, nausea, early satiety, vomiting, heartburn, GI bleeding, and diarrhea. Patients with predominantly muscular involvement may present with symptoms of intestinal obstruction or subobstruction due to thickening of the muscular layer. The rarest form is the predominantly serosal pattern, which is commonly associated with eosinophilic ascites. Less frequently, patients may present with weight loss, steatorrhea, protein‐losing enteropathy, malabsorption, intussusception, and peritonitis.^6^, ^7^ In pediatric patients, growth retardation, failure to thrive, delayed puberty, and amenorrhea may also be observed.^8^

Laboratory tests alone are neither sufficient nor necessary for diagnosing non‐EoE EGIDs; however, they can aid in clinical practice. Approximately 20%–80% of patients show peripheral blood eosinophilia, usually transient, and over 50% exhibit elevated serum IgE levels.^9^, ^10^ Other laboratory abnormalities include anemia, steatorrhea, iron or vitamin deficiencies, hypoalbuminemia, and increased C‐reactive protein (CRP) levels.^9^

While the incidence and prevalence of eosinophilic esophagitis (EoE) are well‐established and have been increasing over the past decade,^11^ precise epidemiological data for non‐EoE EGIDs are lacking.^1^ Nonetheless, it is likely that the rising trend in EoE also applies to non‐EoE EGIDs.^12^ This increasing awareness, coupled with the limited available scientific data, has led to growing interest in non‐EoE EGIDs in recent years.

Additionally, the first international pediatric guidelines for non‐EoE EGIDs were published, offering clinicians diagnostic and therapeutic guidance.^5^ Despite recent guidelines, there is limited data available on pediatric patients to serve as a real‐life reference.^5^ It is worth noting that even in early‐onset forms of EoE (referred to as very early onset EoE, or VEO‐EoE), specific clinical and pathological characteristics have been described compared to later‐onset forms.^13^ However, data regarding early‐onset presentations of non‐EoE EGIDs remain limited in the current literature. Aim of the present study was to characterize non‐EoE EGIDs in a large multicenter Italian pediatric cohort. In addition, we seek to evaluate therapeutic management, endoscopic reassessment, and clinical, endoscopic, and histological responses to treatment. Particular attention was given to identifying the distinctive features of patients with very early‐onset disease.

## 2 METHODS

### 2.1 Ethics statement

The study design was approved by the ethics committee of coordinator center “Comitato Etico Regionale della Toscana Pediatrico n. CET_04/2023” and by ethic committees of all participating centers. The study was conducted according to the criteria set by the declaration of Helsinki.

### 2.2 Study design

A multicenter observational retrospective study was conducted across 12 referral centers from the Italian Society of Pediatric Gastroenterology Hepatology and Nutrition (SIGENP). Children and adolescents below 18 years of age, diagnosed with non‐EoE EGIDs, were retrospectively identified and included in the analysis. Patients diagnosed solely with EoE were excluded.

Other clinically relevant conditions associated with GI mucosal eosinophilia, such as allergic diseases, parasitic infections (e.g., *Helicobacter pylori*, intestinal parasites), drug‐induced reactions, inflammatory bowel diseases (IBDs), and malignancies, infants (under 1 year of age) with proctocolitis, were considered in the differential diagnosis and subsequently excluded.

The diagnosis was established for non‐EoE EGIDs, as recommended by the latest ESPGHAN guidelines.^5^ According to the consensus thresholds, the site‐specific peak eosinophil counts per high‐power field (HPF; 0.27 mm²) were considered as follows: Stomach: ≥30 eos/HPF (≥110 eos/mm²), duodenum: ≥50 eos/HPF (≥185 eos/mm²), terminal ileum: ≥60 eos/HPF (≥220 eos/mm²), cecum and ascending colon: ≥100 eos/HPF (≥370 eos/mm²), Transverse and descending colon: ≥80 eos/HPF (≥300 eos/mm²), rectum and sigmoid colon: ≥60 eos/HPF (≥220 eos/mm²). Clinical, laboratory, endoscopic, and histologic data were retrieved at diagnosis. Subsequently, aiming to evaluate the different management strategies adopted for such conditions, induction and maintenance therapies were retrieved, as well as data on timing and results of any other endoscopic reassessment during the study period.

In addition, we also compared the characteristics of early‐onset forms, defining a cut‐off at 2 years of age with those of later‐onset forms. As previously described for VEO EoE,^13^ we defined as very early onset EGIDs (VEO‐EGIDs) as occurring in children under 2 years of age, and older than 1 year. Consistent with ESPGHAN/NASPGHAN guidelines,^5^ patients under 1 year with hypereosinophilic proctocolitis were excluded.

For the present study, December 31, 2024, was considered the last retrieval date.

### 2.3 Definitions of laboratory parameters and definition of improvement

See Supplemental Digital Content 1.

### 2.4 Statistical analysis

Data were managed and analyzed using IBM SPSS Statistic (version 30th). Categorical variables were described as frequency and percentages. Continuous variables were evaluated for normal distribution using Kolmogorov–Smirnov goodness‐of‐fit test. Normally distributed continuous variables were presented as mean ± standard deviations (SD). Nonnormally distributed variables were presented as medians and interquartile ranges (IQRs). Comparisons among quantitative variables were carried out by the Mann–Whitney *U* test. Analysis of statistical differences among categorical variables was carried out using the *χ* ^2^ or exact Fisher test, when indicated. All statistical tests were two‐sided, and *p* < 0.05 was considered as the statistically significant threshold.

## 3 RESULTS

### 3.1 Clinical symptoms

A total of 86 patients were retrospectively identified, 61 of whom (71%) were male. Median age at diagnosis was 10.5 years (IQR: 8.5). Thirty‐seven patients (40%) had a first‐degree relative with a history of atopy, and 37 patients (40%) had positive tests for aeroallergens or food allergens, with associated atopic comorbidities, such as allergic rhinitis, atopic dermatitis, or asthma.

No patients in this cohort presented with symptoms (i.e., absence of stool and gas passage) suggesting muscular involvement or with signs of serosal involvement (e.g., ascites).

In our cohort, no patient underwent a full‐thickness (transmural) biopsy. Therefore, our evaluation focused exclusively on mucosal involvement.

Abdominal pain was the most common presenting symptom, reported by 56/86 (64.9%) patients. Diarrhea was reported by 33 patients (38.2%) and was significantly more common in those with colonic involvement (15/17, 88.2%, *p* < 0.001). Vomiting, which led to diagnostic endoscopy, was reported in 34/86 (39.4%) patients. Other notable symptoms included dysphagia and food bolus impaction, growth delay, regurgitation, and constipation.

### 3.2 Endoscopic and histological findings

All patients underwent esophagogastroduodenoscopy (EGDS) as part of their diagnostic workup. Additionally, 52 patients underwent ileocolonoscopy, with seven patients who underwent video capsule endoscopy (VCE) as well. No patients underwent single‐ or double‐balloon enteroscopy with biopsies. Baseline characteristics upon GI tract involvement are reported in Table 1.

**Table 1:** Baseline characteristics upon GI tract involvement.

Characteristics	Gastric involvement only (*n* = 11)	Small bowel involvement only (*n* = 19)	Colonic involvement only (*n* = 17)	Multiple GI tracts involved (*n* = 39)	*p*‐value
Male sex (*n*, %)	8 (72.7)	16 (84.2)	9 (52.9)	28 (71.8)	0.228
Age at diagnosis (years, median [IQR])	8.7 (11.4)	12.5 (5.91)	9 (6)	10.3 (10.1)	0.415
Family history of atopy (*n*, %)	4 (36.3)	4 (21)	6 (35.3)	12 (30.7)	0.141
Allergic diathesis (*n*, %)	4 (36.3)	4 (21)	8 (47)	21 (53.8)	0.206
Concurrent EoE (*n*, %)	6 (54.5)	9 (47.4)	12 (70.6)	26 (66.7)	0.467
Symptoms					
Abdominal pain (*n*, %)	7 (63.6)	15 (78.9)	7 (41.2)	27 (69.2)	0.067
Vomiting (*n*, %)	3 (27.3)	7 (36.9)	8 (47.1)	16 (41)	0.772
Diarrhea (*n*, %)	3 (27.3)	6 (31.5)	15 (88.2)	9 (23.1)	<0.001
Growth delay (*n*, %)	4 (36.3)	5 (26.3)	4 (23.5)	13 (33.3)	0.855
Diagnostic delay (months, median [IQR])	13.2 (16.8)	5 (7.9)	5 (42.3)	13 (34)	0.422
Laboratory finding					
Eosinophilia (*n*, %)	5 (45.5)	7 (36.8)	7 (41.2)	24 (61.5)	0.260
Anemia (*n*, %)	1 (9.1)	2 (10.5)	9 (52.9)	8 (20.5)	0.02
Hypoalbuminemia (*n*, %)	1 (9.1)	0 (0)	1 (5.9)	11 (28.2)	0.018
No laboratory abnormalities (*n*, %)	5 (45.5)	11 (57.9)	7 (41.2)	10 (25.6)	0.110
Treatment strategies					
PPI (*n*, %)	10 (90.9)	7 (38.9)	4 (23.5)	17 (43.6)	<0.001
Topical steroids (*n*, %)	7 (63.6)	5 (33.3)	12 (70.6)	21 (53.8)	0.122
Systemic steroids (*n*, %)	0 (0)	2 (10.5)	1 (5.9)	9 (23.1)	0.181
Diet (*n*, %)	3 (13.3)	14 (73.7)	7 (41.2)	23 (58.9)	0.05

Abbreviations: EoE, eosinophilic esophagitis; GI, gastrointestinal; IQR, interquartile range; PPI, proton pump inhibitors.

The most frequently affected GI tract was the stomach, followed by the small bowel. Of the 52 patients who underwent ileocolonoscopy, 21 had colonic involvement. EGDS findings revealed concomitant EoE in 53 patients. Thirty‐nine patients had involvement of more than one GI tract segment.

No statistically significant differences were observed in the distribution of these symptoms based on the involved anatomical tract. The endoscopic characteristics observed in our patients cohort are summarized in Table 2. Mucosal hyperemia was the most common endoscopic finding in patients with EoG, followed by edema. In patients with small bowel involvement, erosions were the predominant finding. Similarly, in EoC, the most common features were erosions and hyperemia.

**Table 2:** Endoscopic characteristics according to gastrointestinal localizzation.

Endoscopic finding	Stomach (*n* = 39)	Small bowel (*n* = 35)	Colon (*n* = 21)	*p*‐value
None (normal)	8 (20.5%)	7 (21%)	6 (21.5%)	0.719
Erosions, ulcers (*n*, %)	35 (89.7%)	23 (65.7%)	9(42.8%)	<0.001
Hyperemia, friability (*n*, %)	10 (25.6)	9 (25.7)	9 (42.8%)	0.312
Edema, nodularity (*n*, %)	23 (59%)	15 (42.8%)	3 (14%)	0.004

Among patients diagnosed with non‐EoE EGIDs, eosinophilic infiltration in the setting of a normal endoscopic macroscopic appearance was observed in 8/39 patients (20.5%) in the stomach, 7/35 (20%) in the small intestine, and 6/21 (28.5%) in the colon.

### 3.3 Peripheral blood abnormalities

An elevated absolute eosinophil count (>500 cell/mm^3^) was observed in 43/86 patients (50%). Low serum albumin levels were reported only in 13/86 patients (15.1%), with a higher frequency observed in those with involvement of more than one GI tract segment (11/39 vs. 2/47, *p* = 0.018). Anemia was reported in 20/86 patients (23.3%) and was more frequently observed in patients with colonic involvement (9/17 vs. 11/69, *p* = 0.02).

### 3.4 VEO‐EGIDs

Twenty‐one patients (24.4%) were diagnosed between 1 and 2 years of age, VEO‐EGIDs.

The main presenting symptoms, inflammatory localization, and laboratory findings in VEO‐EGIDs compared to older patients are summarized in Table 3. In terms of disease location, VEO‐EGIDs were more likely to involve the colon (13/21, 62%) compared to the older group (8/65, 12.3%, *p* < 0.001).

**Table 3:** Characteristics of patients according to age group.

Characteristic	Age < 2 years (*n* = 21)	Age > 2 years (*n* = 65)	*p*‐value
Male sex (*n*, %)	16 (76.2%)	45 (69.2%)	0.541
Family history of atopy (*n*, %)	8 (38.1%)	18 (27.7%)	0.367
Allergic diathesis (*n*, %)	7 (33%)	30 (46.2%)	0.302
Gastric involvement (*n*, %)	5 (23.8%)	34 (52.3%)	0.023
Small bowel involvement (*n*, %)	10 (38.1%)	15 (45.8%)	0.543
Colonic involvement (*n*, %)	13 (62%)	8 (12.3%)	<0.001
Concurrent EoE (*n*, %)	10 (47.6%)	43 (75.4%)	0.002
Multiple sites involved (*n*, %)	6 (28.6%)	25 (38.5%)	0.412
Abdominal pain (*n*, %)	9 (45%)	46 (71.9%)	0.027
Vomiting (*n*, %)	10 (47.6%)	24 (37.5%)	0.411
Diarrhea (*n*, %)	11 (52.4%)	22 (33.8%)	0.120
Growth delay (*n*, %)	8 (28.1%)	18 (38.1%)	0.390
Dysphagia (*n*, %)	1 (4.7%)	19 (29.2%)	0.02
Food bolus impaction (*n*, %)	1 (4.7%)	13 (20%)	0.172
Diagnostic delay (months, median [IQR])	7.8 (1.7‐17.5)	9.6 (2‐30)	0.326
Hypereosinophilia (*n*, %)	10 (47.6%)	33 (50.8%)	0.802
Anemia (*n*, %)	7 (33.3%)	13 (20%)	0.209
Hypoalbuminemia (*n*, %)	5 (23.8%)	8 (12.3%)	0.201
No laboratory abnormalities (*n*, %)	7 (33.3%)	26 (40%)	0.585

Abbreviations: EoE, eosinophilic esophagitis; IQR, interquartile range.

Among patients with VEO EGIDs and colonic involvement, 1/13 (7.6%) also exhibited gastric involvement, and 1/13 (7.6%) had concomitant involvement of the small intestine

In contrast, gastric involvement was significantly less common in VEO‐EGIDs (5/21, 23.8%) compared to older patients (34/65, 52.3%, *p* = 0.023), and esophageal involvement was also significantly less frequent in VEO‐EGIDs (10/21, 47.6%) compared to older patients (43/65, 75.4%, *p* = 0.002). Older patients more commonly presented with abdominal pain (46/65, 71.9%) and dysphagia (19/65, 29.2%) at diagnosis (*p* = 0.027 and *p* = 0.02, respectively). In contrast, diarrhea at diagnosis was more frequently observed in the VEO‐EGIDs group, although not reaching the canonical threshold for statistical significance (11/21, 52.4%, *p* = 0.12).

### 3.5 Treatment outcomes

The first course of therapy induced clinical improvement and symptomatic remission in 43/86 (50%) of patients.

Table 4 presents the clinical, endoscopic, and histological responses to the different therapeutic regimens, stratified by the various segments of the gastrointestinal tract.

**Table 4:** Response to treatment of different non‐EoE EGIDs.

	PPIs	PPIs + topical steroids	PPIs + systemic steroids	PPIs + elimination diet	Topical steroids	Topical steroids + elimination diet	Systemic steroids	Systemic steroids + elimination diet	Elimination diet	PPIs + elimination diet + topical steroids	PPIs + elimination diet + systemic steroids
EoG (tot *n* = 40) *n* (%)	30 (75)	9 (22.5)	5 (12.5)	13 (32.5)	13 (32.5)	5 (12.5)	8 (20)	3 (7.5)	15 (37.5)	4 (10)	3 (7.5)
Clinical Improvement *n* (%)	16 (53,3)	4 (44.4)	5 (100)	8 (61.5)	7 (53.8)	3 (60)	7 (87.5)	3 (100)	10 (66.7)	2 (50)	3 (100)
Endoscopic improvement *n* (%)	8 (26,7)	4 (44.4)	2 (40)	4 (30.8)	5 (38.5)	3 (60)	3 (37.5)	1 (33.3)	6 (40)	2 (50)	1 (33.3)
Histological improvement *n* (%)	8 (26.7)	4 (44.4)	2 (40)	3 (23.1)	5 (38.5)	3 (60)	2 (25)	1 (33.3)	5 (33.3)	2 (50)	1 (33.3)
EoD or EoN (tot *n* = 37) *n* (%)	25 (67.6)	12 (32.4)	6 (16.2)	9 (24.3)	14 (37.8)	2 (5.4)	8 (21.6)	3 (8.1)	13 (35,1)	2 (5,4)	2 (5.4)
Clinical improvement *n* (%)	14 (56)	8 (66.7)	3 (50)	6 (66.7)	8 (57.1)	2 (100)	3 (37.5)	2 (66.7)	9 (69.2)	2 (100)	2 (100)
Endoscopic improvement *n* (%)	8 (32)	6 (50)	2 (33,3)	3 (33,3)	7 (50)	2 (100)	2 (25)	1 (33.3)	6 (46.2)	2 (100)	1 (50)
Histological improvement *n* (%)	10 (40)	6 (50)	3 (50)	3 (33,3)	6 (42.9)	2 (100)	3 (37.5)	1 (33.3)	6 (46.2)	2 (100)	1 (50)
EoC (tot *n* = 28) i(%)	9 (32.1)	4 (14.3)	2 (7.1)	2 (7.1)	12 (42.9)	2 (7.1)	7 (25)	2 (7,1)	7 (25)	0 (0)	1 (3,6)
Clinical improvement *n* (%)	5 (55.6)	2 (50)	2 (100)	2 (100)	8 (66.7)	2 (100)	4 (57.1)	1 (50)	5 (71.4)	0 (0)	1 (100)
Endoscopic improvement *n* (%)	3 (33.3)	2 (50)	0 (0)	0 (0)	6 (50)	2 (100)	1 (14.3)	0 (0)	2 (28.6)	0 (0)	0 (0)
Histological improvement *n* (%)	0 (0)	0 (0)	0 (0)	0 (0)	2 (16,7)	1 (50)	0 (0)	0 (0)	1 (14.3)	0 (0)	0 (0)
Multisite involvement (tot *n* = 4) *n* (%)	2 (50)	0 (0)	1 (25)	1 (25)	0 (0)	0 (0)	1 (25)	0 (0)	1 (25)	0 (0)	0 (0)
Clinical improvement *n* (%)	2 (100)	0 (0)	1 (100)	1 (100)	0 (0)	0 (0)	1 (100)	0 (0)	1 (100)	0 (0)	0 (0)
Endoscopic improvement *n* (%)	0 (0)	0 (0)	0 (0)	0 (0)	0 (0)	0 (0)	0 (0)	0 (0)	0 (0)	0 (0)	0 (0)
Histological improvement *n* (%)	0 (0)	0 (0)	0 (0)	0 (0)	0 (0)	0 (0)	0 (0)	0 (0)	0 (0)	0 (0)	0 (0)

Abbreviations: EoC, eosinophilic colitis; EoD, eosinophilic duodenitis; EoG, eosinophilic gastritis; EoN, eosinophilic enteritis; non‐EoE EGIDs, nonesophageal eosinophilic gastrointestinal disorders; PPI, proton pump inhibitor.

The minimum follow‐up period for these patients was 3 months, with a median follow‐up time of 16 months (IQR: 9–35 months).

#### 3.5.1 Proton pump inhibitors (PPI)

A total of 38/86 (44%) patients received PPIs at diagnosis. The use of PPIs was more prevalent in patients with gastric involvement (10/11, 90.9%, *p* < 0.001), followed by those with small bowel involvement (7/19, 38.9%), colonic involvement (4/17, 23.5%), and multiregional involvement (17/39, 43.6%).

#### 3.5.2 Elimination diet

Dietary therapy, specifically an elimination diet, was started in 47/86 patients (54.5%).

Four out of 47 (8.5%) patients were treated with exclusive enteral nutrition using an amino acid‐based formula, 36/47 patients (76.6%) followed empiric elimination diets, excluding one to three of the most common food allergens (eggs, milk, soy, peanuts, wheat, and fish) in various combinations. In addition, 7/40 patients (17.5%) underwent a six‐food elimination diet, eliminating wheat, eggs, milk, fish, soy, and peanuts. Dietary interventions were particularly common in patients with small bowel involvement (14/19, 73.7%), followed by those with multiple GI tract involvement (23/39, 58.9%), colonic involvement (7/17, 41.2%), and gastric involvement (3/11, 13.3%).

#### 3.5.3 Topical steroids

Budesonide‐coated capsules were administered as a topical corticosteroid, tailored to the anatomical site of disease involvement. Capsules were crushed to target the gastric mucosa, opened to deliver budesonide to the upper small intestine, or swallowed intact to ensure release in the terminal ileum.^14^

Topical steroids were administered to 45/86 patients (52.3%).

Among these, 7/11 (63.6%) had gastric involvement, 5/19 (26.3%) had small bowel involvement, 12/17 (70.6%) had colonic involvement, and 21/39 (53.8%) had involvement of more than one GI tract.

#### 3.5.4 Systemic steroids

Only 12 patients (14%) received systemic steroids, with 9/12 having more extensive involvement.

Endoscopic reassessment was performed in 61/86 patients, with a median time of 5 months (IQR: 7 months) following the initiation of therapy. In these patients, macroscopic remission was observed in 30/61 (49.2%), while histological remission (defined as an eosinophil count below the pathological threshold observed at diagnosis) was achieved in 25/61 (40.9%).

## 4 DISCUSSION

Non‐EoE EGIDs are rare conditions, and their clinical presentation, treatment approaches, and outcomes remain underreported, particularly in pediatric patients. We conducted a survey on behalf of SIGENP, focusing on the distinctive features of exclusively pediatric EGID.

It is established that initial symptoms vary according to the segment of the GI tract involved.^15^ The extend of endoscopic investigation, either upper endoscopy and or lower endoscopy, was guided by the clinical presentation of the patient at the time of diagnosis.^5^

Although the stomach was the most commonly affected area, a majority of patients also had concomitant esophageal involvement. Notably, nearly half of the patients presented with involvement of multiple GI tracts at time of diagnosis.

All patients in the cohort underwent upper endoscopy as part of their diagnostic workup, and more than half of them were also evaluated for involvement of the lower GI tract. Notably, none of our pediatric patients presented with occlusive symptoms suggesting muscular involvement or with signs of serosal involvement.

It can be speculated that, due to the retrospective nature of our data collection and the rarity of EGIDs involving the muscular or serosal layers in pediatric patients, such cases may have gone undiagnosed.

As reported in the literature, non‐EoE EGIDs do not exhibit specific endoscopic characteristics either in the upper GI tract, small bowel, and colon.^16^, ^17^, ^18^, ^19^ However, the identification of mild abnormalities, such as hyperemia, may be of importance for suspecting such condition and gain in an accurate diagnosis.

In our cohort, the most frequent macroscopic findings were erosions when small bowel was involved, whereas being involved the stomach the most frequent macroscopic findings were mucosal hyperemia and nodularity. In contrast, macroscopic abnormalities such as erosions and hyperemia were identified in only a minority of cases in the colon.

It is important to note that, consistent with literature data ^16^, ^17^, ^18^, ^19^ a proportion of patients in our cohort exhibited a macroscopically normal appearance of the colon, small intestine, and stomach with eosinophilic infiltration exceeding the diagnostic threshold.

Analysis of baseline patient characteristics, tract involvement, and age revealed several key findings.

The clinical features were heterogeneous and varied widely. These data confirm previous reports in the literature.^20^, ^21^, ^22^, ^23^ Klein et al. and Talley et al.^20^, ^21^ described nonspecific symptoms such as nausea and abdominal pain as the primary features. Chang et al. extended these findings,^22^ reporting on 59 adult patients with eosinophilic gastroenteritis who exhibited abdominal pain, nausea, and vomiting. Similarly, in a cohort of 28 children with eosinophilic gastric involvement abdominal pain and vomiting were found to be common.^23^ Other retrospective studies^16^, ^18^, ^24^, ^25^ have also reported variable frequencies of abdominal pain, nausea, vomiting, and dysphagia.

In our cohort, abdominal pain was the most common presenting symptom. In contrast, diarrhea, reported as the main symptom, was significantly more common in patients with colonic involvement.

This observation aligns with a retrospective study of 373 patients (both children and adults), that report diarrhea as more frequently seen in patients with colonic involvement than in those with gastric involvement.^26^

It should be noted that dysphagic symptoms in some patients support the coexistence of EoE and isolated colonic involvement.

Regarding laboratory tests, our data showed considerable variability and a lack of specificity. Low albumin levels were observed when more than one GI tract was involved whereas anemia was more frequently reported in patients with colonic involvement. Notably, blood tests were not useful for diagnosis and follow‐up. For example, while half of the patients had an elevated absolute eosinophil count, the other half had normal eosinophil levels despite the presence of mucosal and histological lesions. Similarly, the recent ESPGHAN guidelines^5^ emphasize that peripheral eosinophilia does not correlate with disease severity, and even patients with severe disease may have normal peripheral blood eosinophil counts.

Another notable finding in our data concerns the subgroup of younger children who were diagnosed before the age of 2 (VEO‐EGIDs). In our cohort, most of these patients had colonic involvement. Diarrhea was more commonly observed in this group, while other symptoms such as abdominal pain and dysphagia were less frequent, reflecting less common gastric and esophageal involvement. As suggested by Shoda et al.,^27^ patients with isolated colonic involvement exhibited a distinct clinical pattern compared to those with other non‐EoE‐EGIDs.

Notably, in very early‐onset IBD (VEO‐IBD), colonic involvement also represents the most common site of disease, suggesting a similar pattern of mucosal lesion development in both conditions within this age group.^28^, ^29^, ^30^

Furthermore, laboratory findings indicated that younger patients commonly presented hypoalbuminemia and anemia. Notably, anemia was particularly associated with more frequent colonic involvement.

Our data also highlight challenges in the management of non‐EoE EGIDs. In our cohort, dietary elimination, topical, PPIs too, and, less frequently, systemic steroids treatments were used in various combinations. Despite employing multiple treatment modalities, only half of our patients experienced symptomatic resolution after a first therapeutic course. This is in contrast with other previous studies in which steroid response rates ranged from 80% to 100%.^6^, ^22^, ^23^, ^31^ The lack of a universal response to first treatment course in our cohort is maybe related to the relatively low proportion of patients who received systemic steroids probably due to the fear of potential side effects in children. On the other hand, one might speculate that some of the persistent symptoms could have been partially attributable to functional origin, and, due to the retrospective nature of the study, it was not possible to ascertain such proportion.

It can also be hypothesized that some of the treatments administered were prescribed to address functional symptoms (e.g., dyspepsia) or to prevent adverse effects of steroid therapy, rather than being directly related to disease localization. Notably, some patients—for example, those treated with PPIs with isolated colonic involvement—showed disease remission, even though the therapy was not directly targeted to the site of disease. This observation suggests the possibility of spontaneous disease remission, independent of the treatment administered.

It is also important to note that not all patients who achieved clinical remission also demonstrated macroscopic and histological healing in our cohort even if it is a therapeutic target in the management of non‐EoE EGIDs.^26^

## 5 CONCLUSION

Non‐EoE EGIDs present with nonspecific GI symptoms, which vary according to the segment of the GI tract involved. For instance, colonic involvement was more frequently associated with diarrhea. Younger patients (VEO‐EGIDs) exhibited unique characteristics, particularly in terms of disease localization, with a higher prevalence of colonic involvement. Laboratory tests were not consistently useful for diagnosis or monitoring; however, certain abnormalities, such as anemia and hypoalbuminemia, were more commonly observed in patients with colonic disease.

Once the diagnosis is established, non‐EoE EGIDs remains difficult to treat, with considerable variability in management and treatment outcomes.

This study has some limitations, including its retrospective design, which may introduce recall bias and limit the available data. However, the data collected from 86 pediatric patients from 12 pediatric gastroenterology centers provides a reliable snapshot of the management of this complex condition. The latest ESPGHAN guidelines^5^ offer diagnostic algorithms and treatment strategies. Larger, prospective future studies are needed to determine the effect of the implementation of such guidelines for Non‐EoE EGIDs.

## CONFLICT OF INTEREST STATEMENT

The authors declare no conflict of interest.

## Supporting information


**Supplemental Digital Content 1**: Definitions of Laboratory Parameters. Definition of Improvement.
