# Supplementary material for: Characterization of pediatric eosinophilic gastrointestinal disorders beyond eosinophilic esophagitis in a nationwide cohort
Source: J Pediatr Gastroenterol Nutr. 2025 Nov 19;82(2):531–40. doi: 10.1002/jpn3.70282 (PMC12864182; doi:10.1002/jpn3.70282)
Supplement: Supplementary file 1 — Supplemental Digital Content 1: Definitions of Laboratory Parameters. Definition of Improvement. [file JPN3-82-531-s001.docx]

**Definitions of Laboratory Parameters**

Anemia was defined, based on World Health Organization (WHO) criteria, as hemoglobin concentration <11.5 g/dL for children aged 1–4 years, <11.5 g/dL for those aged 5–11 years, <12.0 g/dL for children aged 12–14 years, <12.0 g/dL for females aged 15–18 years, and <13.0 g/dL for males aged 15–18 years [1]. Iron deficiency was defined as serum ferritin <15 ng/mL in the absence of inflammation, or <30 ng/mL when C-reactive protein (CRP) was >5 mg/L, according to WHO and ESPGHAN recommendations [1,2]. Hypereosinophilia was defined as an absolute eosinophil count >500 cells/μL, consistent with American Academy of Pediatrics (AAP) guidance [3]. Hypoalbuminemia was defined as serum albumin <3.5 g/dL, based on standard pediatric reference ranges [4].

**Definition of Improvement**

Clinical response was defined as the resolution of previously reported symptoms attributable to EoE, including, when present, vomiting, regurgitation, dysphagia, growth delay, and abdominal pain.
Endoscopic remission was defined as the healing of pathological macroscopic findings described at diagnostic endoscopy, specifically mucosal nodularity, erosions, ulcers, edema, erythema, and friability.
Histological response was defined as a reduction in peak eosinophil count below the pathological threshold defined by the ESPGHAN/NASPGHAN guidelines^(5)^ for Non-esophageal eosinophilic gastrointestinal disorders (Non-EoE EGIDs), as observed at diagnosis.

1. World Health Organization. Haemoglobin concentrations for the diagnosis of anaemia and assessment of severity. Geneva: WHO; 2011. Report No.: WHO/NMH/NHD/MNM/11.1.

2. Pimenta AM, Galvão TF, Silva MT, Araujo ME, Pereira MG. Ferritin levels and risk of iron deficiency in children and adolescents: a systematic review. Rev Paul Pediatr. 2020;38:e2018113. doi:10.1590/1984-0462/2020/38/2018113.

3. Simon HU, Rothenberg ME, Bochner BS, Weller PF, Wardlaw AJ, Wechsler ME, et al. Refining the definition of hypereosinophilic syndrome. J Allergy Clin Immunol. 2010;126(1):45–9. doi:10.1016/j.jaci.2010.03.028.

4. Shaw V, Lawson M, editors. Clinical Paediatric Dietetics. 4th ed. Oxford: Wiley-Blackwell; 2014. Chapter 4: Biochemistry and nutrition

1. Papadopoulou A, Amil-Dias J, Auth MK-H, et al. Joint ESPGHAN/NASPGHAN Guidelines on Childhood Eosinophilic Gastrointestinal Disorders Beyond Eosinophilic Esophagitis. *J Pediatr Gastroenterol Nutr* 2024; 78:122–152. https://doi.org/10.1097/MPG.0000000000003877
